# Caffeinium bis­ulfate monohydrate

**DOI:** 10.1107/S1600536811031540

**Published:** 2011-08-11

**Authors:** C. Vincent Jerin, S. Athimoolam

**Affiliations:** a Department of Physics, University College of Engineering Nagercoil, Anna University of Technology Tirunelveli, Nagercoil 629 004, India

## Abstract

In the title compound (systematic name: 1,3,7-trimethyl-2,6-dioxo-7*H*-purin-9-ium hydrogen sulfate monohydrate), C_8_H_11_N_4_O_2_^+^·HSO_4_^−^·H_2_O, the crystal packing is stabilized through N—H⋯O and O—H⋯O hydrogen bonds.

## Related literature

For background to caffeine, see: Benowitz (1990 ▶); Smith (2002 ▶); Griesser & Burger (1995 ▶); Bothe & Cammenga (1980 ▶); Edwards *et al.* (1997 ▶); Sutor (1958 ▶); Trask *et al.* (2005 ▶). For hydrogen-bond motifs, see: Etter *et al.* (1990 ▶).
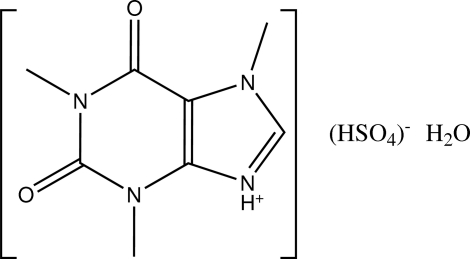


## Experimental

### 

#### Crystal data

C_8_H_11_N_4_O_2_^+^·HSO_4_^−^·H_2_O
*M* *_r_* = 310.29
Monoclinic, 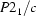

*a* = 9.8296 (10) Å
*b* = 6.2879 (6) Å
*c* = 21.340 (2) Å
β = 90.788 (2)°
*V* = 1318.8 (2) Å^3^
*Z* = 4
Mo *K*α radiation
μ = 0.29 mm^−1^
*T* = 293 K
0.21 × 0.18 × 0.13 mm

#### Data collection

Bruker SMART APEX CCD area-detector diffractometer
11981 measured reflections
2317 independent reflections
2200 reflections with *I* > 2σ(*I*)
*R*_int_ = 0.020

#### Refinement

*R*[*F* ^2^ > 2σ(*F* ^2^)] = 0.039
*wR*(*F* ^2^) = 0.111
*S* = 1.08
2317 reflections
201 parameters
3 restraints
H atoms treated by a mixture of independent and constrained refinement
Δρ_max_ = 0.43 e Å^−3^
Δρ_min_ = −0.38 e Å^−3^

### 

Data collection: *SMART* (Bruker, 2001 ▶); cell refinement: *SAINT* (Bruker, 2001 ▶); data reduction: *SAINT*; program(s) used to solve structure: *SHELXTL/PC* (Sheldrick, 2008 ▶); program(s) used to refine structure: *SHELXTL/PC*; molecular graphics: *PLATON* (Spek, 2009 ▶); software used to prepare material for publication: *SHELXTL/PC*.

## Supplementary Material

Crystal structure: contains datablock(s) global, I. DOI: 10.1107/S1600536811031540/bt5603sup1.cif

Structure factors: contains datablock(s) I. DOI: 10.1107/S1600536811031540/bt5603Isup2.hkl

Supplementary material file. DOI: 10.1107/S1600536811031540/bt5603Isup3.cml

Additional supplementary materials:  crystallographic information; 3D view; checkCIF report

## Figures and Tables

**Table 1:** Hydrogen-bond geometry (Å, °)

*D*—H⋯*A*	*D*—H	H⋯*A*	*D*⋯*A*	*D*—H⋯*A*
N1—H1*N*⋯O13^i^	0.88 (2)	1.83 (2)	2.709 (2)	174 (2)
O14—H14⋯O1*W*^ii^	0.88 (3)	1.60 (4)	2.479 (3)	171 (3)
O1*W*—H1*W*⋯O13^iii^	0.94 (1)	1.82 (1)	2.742 (2)	168 (4)
O1*W*—H2*W*⋯O11	0.94 (1)	1.98 (3)	2.711 (2)	133 (3)

Symmetry codes: (i) 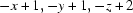; (ii) 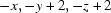; (iii) 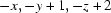.

**Fig. 1.**
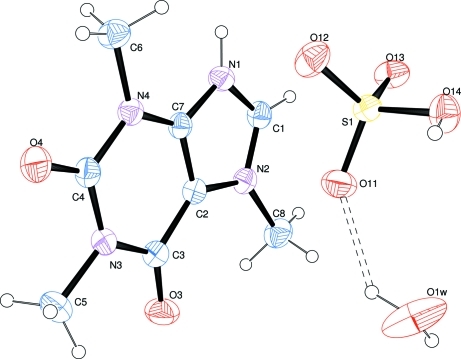
The molecular structure of the title compound with atom numbering scheme and 50% probability displacement ellipsoids. H-bonds are shown as dashed lines.

**Fig. 2.**
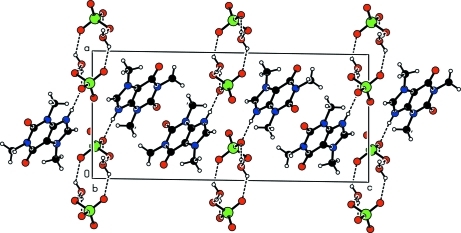
Packing diagram of the title compound viewed down the b-axis. H-bonds are shown as dashed lines.

## supplementary crystallographic information

### Comment

Caffeine is an alkaloid and structurally identified as 1,3,7-trimethylxanthine. It is one of several xanthine derivatives which occur naturally in cofee beans, tea leaves, kola nuts and cocoa beans. It is the most widely consumed stimulant drug in the world (Benowitz, 1990). Caffeine is a central nervous system stimulant and a smooth muscle relaxant, and is commonly employed as a formulation additive to analgesic remedies. Moderate consumption of caffeine increases alertness and reduces fatigues (Smith, 2002). Apart from the above, caffeine is a model pharmaceutical compound that is known to exhibit instability with respect to humidity, with the formation of a crystalline nonstoichiometric hydrate (Griesser & Burger, 1995). Its solid-state properties have been widely investigated; it is known to occur in two anhydrous crystal froms (α, β), one crystalline nonstoichiometric hydrate (Bothe & Cammenga, 1980) and a number of simple cocrystals and salts (Trask *et al.*, 2005). The crystal structure of the hydrated form was determined a long time ago (Sutor, 1958) and confirmed recently (Edwards *et al.*, 1997). In the present work, caffeine was treated with sulfuric acid and the structure of the title compound, (I), is reported here.

The asymmetric part of (I) contains a caffeinium cation, bisulfate anion and a lattice water molecule (Fig. 1). The protonation on the N site of the cation is confirmed from C—N bond distances and C—N—C bond angle. The presence of H atom in one of the O atoms of the bisulfate anion is confirmed from the asymmetric S—O bond distances. This ascertain the bisulfate nature of the anion. The crystal packing is stabilized through N–H···O and O–H···O hydrogen bonds (Table 1; Fig. 2). Anions are dimerized themselves through lattice water molecule and making two adjacent ring *R*_4_^4^(12) motifs around the inversion centeres of the unit cell (Etter *et al.*, 1990). Further, cations are linked to this anionic dimers through another N–H···O hydrogen bond.

### Experimental

The title compound was crystallized from an aqueous mixture containing caffeine and sulfuric acid in the stoichiometric ratio of 1:1 at room temperature by slow evaporation technique.

### Refinement

All the H atoms, except the H atoms involved in the hydrogen bonds, were positioned geometrically and refined by the riding model approximation with d(C—H) = 0.93 Å and *U*_iso_(H)= 1.2 *U*_eq_(C) for aromatic H and d(C—H) = 0.96 Å and *U*_iso_(H)= 1.5 *U*_eq_(C) for methyl H. The H atoms of the –NH group of the cation and water molecule were located from difference fourier map and refined isotropically. The O-H distances of the water molecule are restrained to 0.95 (1)Å and the H···H distance to 1.64 (10)Å.

### Crystal data

**Table:**

C_8_H_11_N_4_O_2_^+^·HSO_4_^−^·H_2_O	*F*(000) = 648
*M**_r_* = 310.29	*D*_x_ = 1.563 Mg m^−^^3^*D*_m_ = 1.55 (1) Mg m^−^^3^*D*_m_ measured by flotation technique using a liquid-mixture of xylene and bromoform
Monoclinic, *P*2_1_/*c*	Mo *K*α radiation, λ = 0.71073 Å
Hall symbol: -P 2ybc	Cell parameters from 2986 reflections
*a* = 9.8296 (10) Å	θ = 2.1–24.4°
*b* = 6.2879 (6) Å	µ = 0.29 mm^−^^1^
*c* = 21.340 (2) Å	*T* = 293 K
β = 90.788 (2)°	Block, colourless
*V* = 1318.8 (2) Å^3^	0.21 × 0.18 × 0.13 mm
*Z* = 4	

### Data collection

**Table:**

Bruker SMART APEX CCD area-detector diffractometer	2200 reflections with *I* > 2σ(*I*)
Radiation source: fine-focus sealed tube	*R*_int_ = 0.020
graphite	θ_max_ = 25.0°, θ_min_ = 1.9°
ω scans	*h* = −11→11
11981 measured reflections	*k* = −7→7
2317 independent reflections	*l* = −25→25

### Refinement

**Table:**

Refinement on *F*^2^	Secondary atom site location: difference Fourier map
Least-squares matrix: full	Hydrogen site location: inferred from neighbouring sites
*R*[*F*^2^ > 2σ(*F*^2^)] = 0.039	H atoms treated by a mixture of independent and constrained refinement
*wR*(*F*^2^) = 0.111	*w* = 1/[σ^2^(*F*_o_^2^) + (0.0659*P*)^2^ + 0.5199*P*] where *P* = (*F*_o_^2^ + 2*F*_c_^2^)/3
*S* = 1.08	(Δ/σ)_max_ = 0.001
2317 reflections	Δρ_max_ = 0.43 e Å^−^^3^
201 parameters	Δρ_min_ = −0.38 e Å^−^^3^
3 restraints	Extinction correction: *SHELXTL/PC* (Sheldrick, 2008), Fc^*^=kFc[1+0.001xFc^2^λ^3^/sin(2θ)]^-1/4^
Primary atom site location: structure-invariant direct methods	Extinction coefficient: 0.030 (3)

### Special details

**Table:**

Geometry. All e.s.d.'s (except the e.s.d. in the dihedral angle between two l.s. planes) are estimated using the full covariance matrix. The cell e.s.d.'s are taken into account individually in the estimation of e.s.d.'s in distances, angles and torsion angles; correlations between e.s.d.'s in cell parameters are only used when they are defined by crystal symmetry. An approximate (isotropic) treatment of cell e.s.d.'s is used for estimating e.s.d.'s involving l.s. planes.
Refinement. Refinement of *F*^2^ against ALL reflections. The weighted *R*-factor *wR* and goodness of fit *S* are based on *F*^2^, conventional *R*-factors *R* are based on *F*, with *F* set to zero for negative *F*^2^. The threshold expression of *F*^2^ > σ(*F*^2^) is used only for calculating *R*-factors(gt) *etc*. and is not relevant to the choice of reflections for refinement. *R*-factors based on *F*^2^ are statistically about twice as large as those based on *F*, and *R*- factors based on ALL data will be even larger.

### Fractional atomic coordinates and isotropic or equivalent isotropic displacement parameters (Å^2^)

**Table:**

	*x*	*y*	*z*	*U*_iso_*/*U*_eq_	
C1	0.36935 (19)	0.2793 (3)	0.91192 (9)	0.0440 (4)	
H1	0.3794	0.1702	0.9410	0.053*	
C2	0.28869 (17)	0.4803 (3)	0.83632 (8)	0.0400 (4)	
C3	0.21533 (19)	0.5681 (3)	0.78425 (9)	0.0462 (5)	
C4	0.39035 (19)	0.8556 (3)	0.78816 (8)	0.0429 (4)	
C5	0.2076 (2)	0.8651 (4)	0.71053 (11)	0.0653 (6)	
H5A	0.1993	1.0144	0.7191	0.098*	
H5B	0.1187	0.8054	0.7035	0.098*	
H5C	0.2616	0.8448	0.6739	0.098*	
C6	0.5833 (2)	0.8438 (3)	0.86283 (10)	0.0516 (5)	
H6A	0.5826	0.8389	0.9078	0.077*	
H6B	0.5927	0.9886	0.8493	0.077*	
H6C	0.6583	0.7614	0.8478	0.077*	
C7	0.40523 (17)	0.5697 (3)	0.85864 (8)	0.0373 (4)	
C8	0.1474 (2)	0.1599 (4)	0.86777 (11)	0.0580 (5)	
H8A	0.1670	0.0271	0.8882	0.087*	
H8B	0.1234	0.1342	0.8247	0.087*	
H8C	0.0729	0.2283	0.8883	0.087*	
N1	0.45565 (16)	0.4425 (2)	0.90488 (7)	0.0417 (4)	
N2	0.26785 (15)	0.2975 (2)	0.87113 (7)	0.0418 (4)	
N3	0.27344 (16)	0.7589 (3)	0.76434 (7)	0.0444 (4)	
N4	0.45593 (15)	0.7565 (2)	0.83794 (7)	0.0395 (4)	
O3	0.11562 (16)	0.4920 (3)	0.75848 (8)	0.0699 (5)	
O4	0.43242 (15)	1.0219 (2)	0.76720 (7)	0.0582 (4)	
H1N	0.533 (2)	0.454 (4)	0.9257 (10)	0.051 (6)*	
S1	0.25373 (4)	0.72796 (7)	1.01018 (2)	0.0398 (2)	
O11	0.16152 (15)	0.6931 (3)	0.95835 (7)	0.0600 (4)	
O12	0.36818 (15)	0.8565 (2)	0.99415 (8)	0.0623 (4)	
O13	0.29697 (14)	0.5271 (2)	1.03835 (7)	0.0521 (4)	
O14	0.17650 (16)	0.8381 (3)	1.06289 (7)	0.0580 (4)	
H14	0.152 (3)	0.966 (6)	1.0503 (14)	0.092 (10)*	
O1W	−0.10677 (19)	0.7918 (3)	0.96137 (17)	0.1136 (10)	
H1W	−0.162 (3)	0.670 (4)	0.9608 (17)	0.130 (13)*	
H2W	−0.035 (3)	0.729 (6)	0.9394 (17)	0.139 (16)*	

### Atomic displacement parameters (Å^2^)

**Table:**

	*U*^11^	*U*^22^	*U*^33^	*U*^12^	*U*^13^	*U*^23^
C1	0.0478 (10)	0.0389 (10)	0.0454 (10)	0.0031 (8)	0.0057 (8)	0.0045 (8)
C2	0.0389 (9)	0.0416 (10)	0.0397 (9)	−0.0008 (7)	0.0042 (7)	0.0005 (7)
C3	0.0414 (10)	0.0537 (11)	0.0435 (10)	−0.0022 (8)	0.0015 (8)	0.0016 (8)
C4	0.0453 (10)	0.0436 (10)	0.0401 (9)	0.0030 (8)	0.0079 (7)	0.0026 (8)
C5	0.0641 (13)	0.0766 (16)	0.0550 (12)	0.0001 (12)	−0.0103 (10)	0.0224 (12)
C6	0.0469 (10)	0.0471 (11)	0.0606 (12)	−0.0079 (9)	−0.0030 (9)	0.0005 (9)
C7	0.0384 (9)	0.0370 (9)	0.0367 (8)	0.0023 (7)	0.0052 (7)	−0.0009 (7)
C8	0.0530 (12)	0.0536 (12)	0.0675 (13)	−0.0146 (10)	0.0047 (10)	0.0054 (10)
N1	0.0404 (8)	0.0423 (8)	0.0422 (8)	0.0026 (7)	−0.0022 (6)	0.0030 (6)
N2	0.0414 (8)	0.0392 (8)	0.0450 (8)	−0.0028 (6)	0.0057 (6)	0.0021 (6)
N3	0.0427 (8)	0.0515 (10)	0.0389 (8)	0.0021 (7)	0.0011 (7)	0.0071 (7)
N4	0.0398 (8)	0.0387 (8)	0.0401 (8)	−0.0023 (6)	0.0021 (6)	0.0007 (6)
O3	0.0569 (9)	0.0816 (12)	0.0706 (10)	−0.0200 (8)	−0.0213 (8)	0.0154 (9)
O4	0.0639 (9)	0.0486 (8)	0.0622 (9)	−0.0070 (7)	0.0035 (7)	0.0160 (7)
S1	0.0371 (3)	0.0369 (3)	0.0455 (3)	−0.00028 (17)	−0.00039 (19)	0.00490 (17)
O11	0.0530 (8)	0.0711 (10)	0.0555 (8)	0.0094 (8)	−0.0120 (7)	−0.0037 (7)
O12	0.0524 (8)	0.0540 (9)	0.0805 (10)	−0.0106 (7)	0.0090 (7)	0.0128 (8)
O13	0.0465 (7)	0.0392 (7)	0.0704 (9)	0.0000 (6)	−0.0095 (6)	0.0100 (6)
O14	0.0686 (10)	0.0524 (9)	0.0534 (8)	0.0080 (8)	0.0123 (7)	0.0020 (7)
O1W	0.0492 (10)	0.0435 (10)	0.248 (3)	−0.0019 (8)	−0.0118 (15)	0.0082 (14)

### Geometric parameters (Å, °)

**Table:**

C1—N2	1.320 (3)	C6—H6B	0.9600
C1—N1	1.341 (3)	C6—H6C	0.9600
C1—H1	0.9300	C7—N4	1.352 (2)
C2—C7	1.357 (3)	C7—N1	1.359 (2)
C2—N2	1.385 (2)	C8—N2	1.468 (2)
C2—C3	1.427 (3)	C8—H8A	0.9600
C3—O3	1.215 (2)	C8—H8B	0.9600
C3—N3	1.398 (3)	C8—H8C	0.9600
C4—O4	1.212 (2)	N1—H1N	0.88 (2)
C4—N4	1.383 (2)	S1—O12	1.430 (1)
C4—N3	1.390 (3)	S1—O11	1.437 (1)
C5—N3	1.471 (2)	S1—O13	1.459 (1)
C5—H5A	0.9600	S1—O14	1.531 (2)
C5—H5B	0.9600	O14—H14	0.88 (3)
C5—H5C	0.9600	O1W—H1W	0.94 (1)
C6—N4	1.461 (2)	O1W—H2W	0.94 (1)
C6—H6A	0.9600		
			
N2—C1—N1	109.47 (16)	N2—C8—H8A	109.5
N2—C1—H1	125.3	N2—C8—H8B	109.5
N1—C1—H1	125.3	H8A—C8—H8B	109.5
C7—C2—N2	106.62 (15)	N2—C8—H8C	109.5
C7—C2—C3	121.88 (17)	H8A—C8—H8C	109.5
N2—C2—C3	131.41 (17)	H8B—C8—H8C	109.5
O3—C3—N3	122.05 (18)	C1—N1—C7	107.88 (16)
O3—C3—C2	126.57 (19)	C1—N1—H1N	123.5 (15)
N3—C3—C2	111.37 (16)	C7—N1—H1N	128.5 (15)
O4—C4—N4	120.96 (18)	C1—N2—C2	108.04 (15)
O4—C4—N3	121.79 (17)	C1—N2—C8	125.67 (17)
N4—C4—N3	117.24 (16)	C2—N2—C8	126.03 (17)
N3—C5—H5A	109.5	C4—N3—C3	127.13 (16)
N3—C5—H5B	109.5	C4—N3—C5	116.08 (17)
H5A—C5—H5B	109.5	C3—N3—C5	116.73 (17)
N3—C5—H5C	109.5	C7—N4—C4	118.19 (16)
H5A—C5—H5C	109.5	C7—N4—C6	121.72 (16)
H5B—C5—H5C	109.5	C4—N4—C6	119.85 (16)
N4—C6—H6A	109.5	O12—S1—O11	113.08 (10)
N4—C6—H6B	109.5	O12—S1—O13	111.21 (9)
H6A—C6—H6B	109.5	O11—S1—O13	111.21 (9)
N4—C6—H6C	109.5	O12—S1—O14	108.64 (10)
H6A—C6—H6C	109.5	O11—S1—O14	108.70 (9)
H6B—C6—H6C	109.5	O13—S1—O14	103.50 (9)
N4—C7—C2	123.95 (17)	S1—O14—H14	109 (2)
N4—C7—N1	128.05 (17)	H1W—O1W—H2W	95 (3)
C2—C7—N1	107.97 (16)		
			
C7—C2—C3—O3	174.8 (2)	O4—C4—N3—C3	179.76 (17)
N2—C2—C3—O3	−1.3 (3)	N4—C4—N3—C3	−1.5 (3)
C7—C2—C3—N3	−4.5 (2)	O4—C4—N3—C5	2.6 (3)
N2—C2—C3—N3	179.39 (18)	N4—C4—N3—C5	−178.66 (17)
N2—C2—C7—N4	−177.11 (15)	O3—C3—N3—C4	−176.83 (19)
C3—C2—C7—N4	5.9 (3)	C2—C3—N3—C4	2.5 (3)
N2—C2—C7—N1	1.3 (2)	O3—C3—N3—C5	0.3 (3)
C3—C2—C7—N1	−175.68 (16)	C2—C3—N3—C5	179.64 (18)
N2—C1—N1—C7	1.0 (2)	C2—C7—N4—C4	−4.5 (3)
N4—C7—N1—C1	176.86 (17)	N1—C7—N4—C4	177.41 (16)
C2—C7—N1—C1	−1.4 (2)	C2—C7—N4—C6	−178.87 (17)
N1—C1—N2—C2	−0.2 (2)	N1—C7—N4—C6	3.1 (3)
N1—C1—N2—C8	−174.58 (18)	O4—C4—N4—C7	−179.05 (17)
C7—C2—N2—C1	−0.7 (2)	N3—C4—N4—C7	2.2 (2)
C3—C2—N2—C1	175.91 (19)	O4—C4—N4—C6	−4.6 (3)
C7—C2—N2—C8	173.66 (17)	N3—C4—N4—C6	176.66 (16)
C3—C2—N2—C8	−9.8 (3)		

### Hydrogen-bond geometry (Å, °)

**Table:**

*D*—H···*A*	*D*—H	H···*A*	*D*···*A*	*D*—H···*A*
N1—H1N···O13^i^	0.88 (2)	1.83 (2)	2.709 (2)	174 (2)
O14—H14···O1W^ii^	0.88 (3)	1.60 (4)	2.479 (3)	171 (3)
O1W—H1W···O13^iii^	0.94 (1)	1.82 (1)	2.742 (2)	168 (4)
O1W—H2W···O11	0.94 (1)	1.98 (3)	2.711 (2)	133 (3)

Symmetry codes: (i) −*x*+1, −*y*+1, −*z*+2; (ii) −*x*, −*y*+2, −*z*+2; (iii) −*x*, −*y*+1, −*z*+2.
